# State‐of‐the‐art consensus on non‐transvenous implantable cardioverter‐defibrillator therapy

**DOI:** 10.1002/clc.23432

**Published:** 2020-08-14

**Authors:** Christoph Schukro, David Santer, Günther Prenner, Markus Stühlinger, Martin Martinek, Alexander Teubl, Deddo Moertl, Stefan Schwarz, Michael Nürnberg, Lukas Fiedler, Robert Hatala, Cesar Khazen

**Affiliations:** ^1^ Department of Internal Medicine II, Division of Cardiology Medical University of Vienna Vienna Austria; ^2^ Department of Cardiac Surgery University Hospital Basel Basel Switzerland; ^3^ Department of Internal Medicine, Division of Cardiology Medical University of Graz Graz Austria; ^4^ Department of Internal Medicine III Medical University of Innsbruck Innsbruck Austria; ^5^ Department of Internal Medicine I Ordensklinikum Linz Elisabethinen Hospital Linz Austria; ^6^ Department of Internal Medicine III Wiener Neustadt State Hospital Wiener Neustadt Austria; ^7^ Department of Internal Medicine III University Hospital St. Pölten Austria; ^8^ Department of Internal Medicine I Kepler University Hospital Linz Linz Austria; ^9^ Department of Internal Medicine III Wilhelminen Hospital Vienna Vienna Austria; ^10^ Department of Arrhythmias and Cardiac Pacing National Institute of Cardiovascular Diseases and Slovak Medical University Bratislava Slovakia; ^11^ Department of Surgery, Division of Cardiac Surgery Medical University of Vienna Vienna Austria

**Keywords:** consensus document, non‐transvenous implantable cardioverter defibrillator, recommendations, subcutaneous implantable cardioverter defibrillator

## Abstract

Within the last decade, implantable cardioverter‐defibrillator (ICD) systems with non‐transvenous leads were developed in order to minimize complications related to the cardiovascular position of transvenous ICD leads. This national expert consensus gives an overview of potential indications for the implantation of non‐transvenous ICD systems, and provides specific recommendations for implantation, follow‐up, and complication management in patients with subcutaneous ICD. Regarding particular issues like the necessity for shock efficacy testing, or the clinical outcome as compared to transvenous ICD, randomized data are expected in the near future.

## 1 INTRODUCTION

The very first implantable cardioverter‐defibrillator (ICD) devices have been developed almost 50 years ago.^1^ Implanted subcutaneously in the abdomen, these devices were able to deliver high‐energy shocks through a lead with an epicardially sewed patch‐electrode in case of ventricular fibrillation. Because of a high incidence of patch‐electrode fractures as well as a higher perioperative risk, these epicardial systems were replaced by transvenous ICD (TV‐ICD) with endocardial leads in the 1990's. Although these systems were easier to implant and even enabled painless anti‐tachycardia pacing (ATP) for termination of ventricular tachycardia (VT), recent data showed that most complications of TV‐ICD, needing surgical revision, were mainly associated with transvenous lead failure, especially due to systemic infections, thrombosis or lead fracture.^2^ In order to minimize such complications related to the cardiovascular position of transvenous leads, ICD systems with “extracardiac” leads were developed within the last decade. Whereas the first “extravascular ICD” (EV‐ICD) was implanted last year,^3^ the “subcutaneous ICD” (S‐ICD) was launched about 10 years ago.^4^

This national expert consensus aims to summarize the state‐of‐the‐art on non‐transvenous ICD (NTV‐ICD) therapy, regarding indications, technical screening, implantation issues, device follow‐up, and the management of complications related to this therapy. Classes of recommendation (I, IIa, IIb, III) and levels of evidence (A, B, C) are consistent with the grading in all recent ESC guidelines.

## 2 NTV‐ICD SYSTEMS

### 2.1 Subcutaneous ICD

In general, the current S‐ICD system consist of two parts: a “shock‐only” defibrillator device, implanted between the muscles *serratus anterior* and *latissimus dorsi* in a left lateral position, and an exclusively subcutaneous lead for both, ECG‐detection, and defibrillation. The lead tip is normally positioned at the level of the second intercostal space of the left sternal border. This system has three electrodes for ECG‐detection: one located at the tip and— separated by a shock coil of 8 cm in length— another in the middle of the lead, as well as the device itself. The vectors resulting from these electrodes (referred to as: *primary* = between middle electrode and device, *secondary* = between tip electrode and device, *alternative* = between both electrodes) are used for ECG detection. The delivered shock energy (80 J) is twice as high as the one delivered by standard transvenous ICD devices with conventional endocardial leads.

### 2.2 Extravascular ICD

In principle, the EV‐ICD arrangement is similar to the S‐ICD: a defibrillator implanted in a left lateral thoracic position with a lead close to the sternum. The decisive difference is the strictly retrosternal implantation of an S‐shaped lead in the mediastinal space. This concept was designed to provide effective bradycardia pacing as well as ATP like TV‐ICD systems, by means of a closer proximity of the EV‐ICD lead to the heart. Due to lack of direct contact with cardiac tissue, sensing properties are lower and pacing thresholds substantially higher than in TV‐ICD systems.

## 3 DECISION‐MAKING FOR IMPLANTATION OF NTV‐ICD

### 3.1 Underlying diseases

**FIGURE 1:**
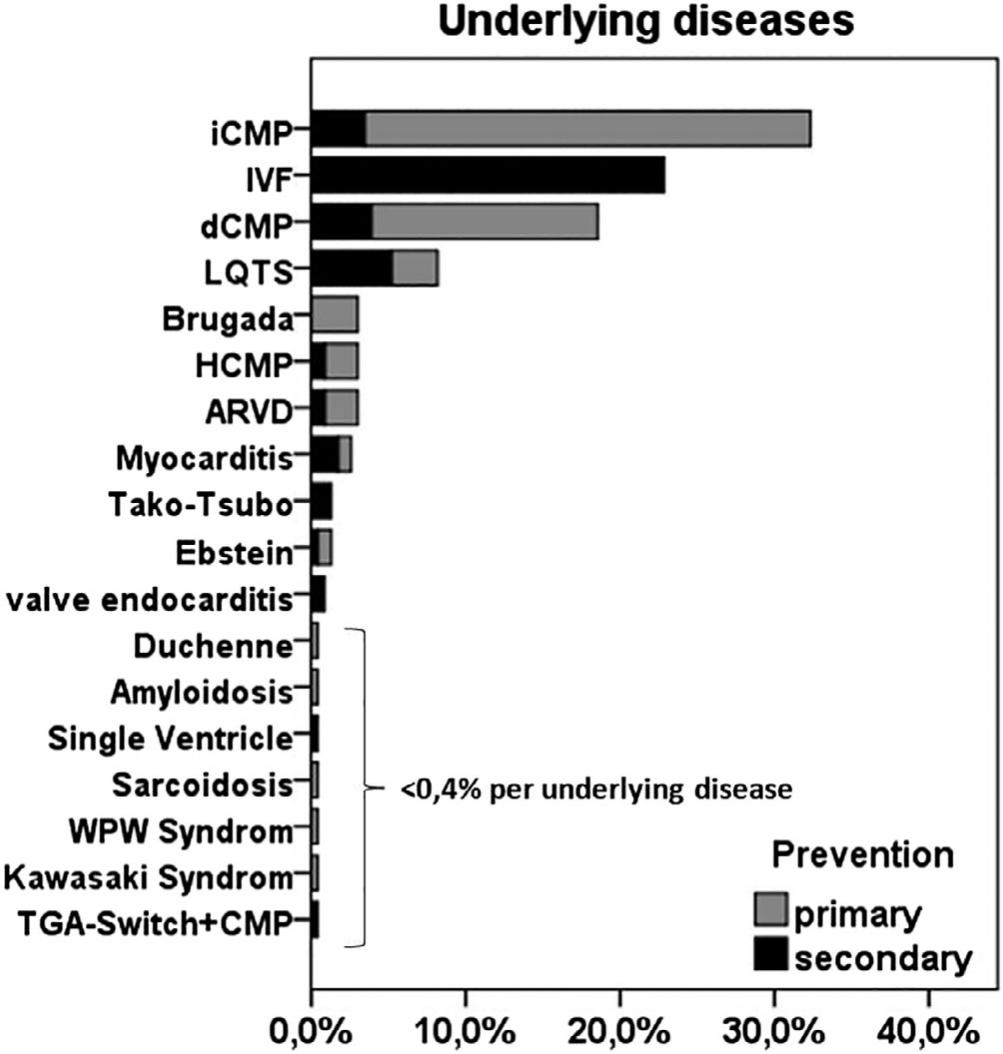
Most frequent underlying diseases in S‐ICD patients depending on primary and secondary prevention. ARVD, arrhythmogenic ventricular dysplasia; dCMP, dilated cardiomyopathy; HCMP, hypertrophic cardiomyopathy; iCMP, ischemic cardiomyopathy (ischemic LV dysfunction); IVF, idiopathic ventricular fibrillation; LQTS, long‐QT syndrome; S‐ICD, subcutaneous implantable cardioverter‐defibrillator; TGA, transposition of great arteries

Generally, the underlying diseases in patients with NTV‐ICD do not differ from the ones in patients with TV‐ICD. In our recently published nationwide multicenter registry, the most frequent underlying diseases of in our cohort were ischemic left ventricular dysfunction (32.0%), idiopathic ventricular fibrillation (IVF) (22.6%), dilated (nonischemic) CMP (17.3%), and long‐QT‐syndrome (8.1%) (Figure 1).^5^

### 3.2 Indications for implantation

**FIGURE 2:**
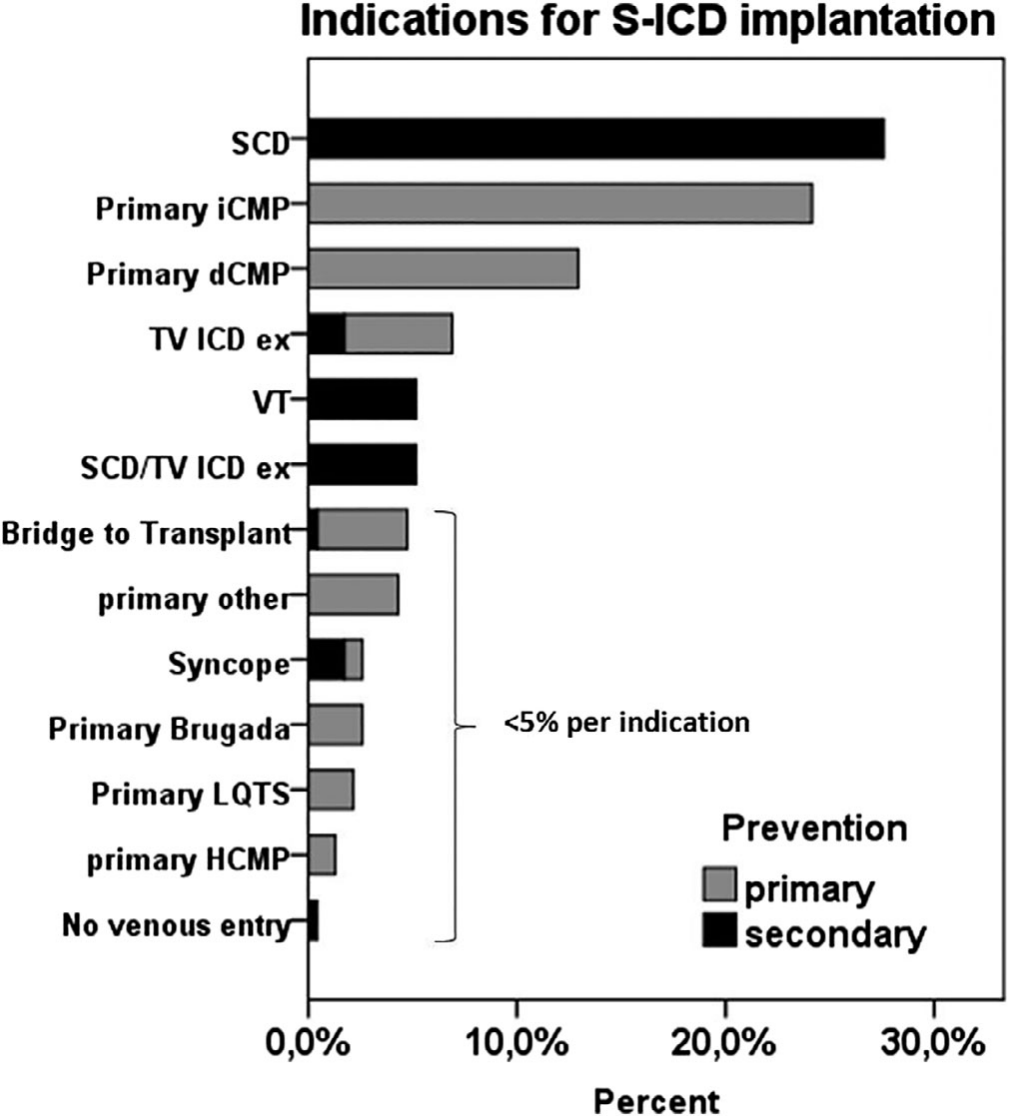
Most frequent indications for S‐ICD implantation depending on primary and secondary prevention. dCMP, dilated cardiomyopathy; HCMP, hypertrophic cardiomyopathy; iCMP: ischemic cardiomyopathy (ischemic LV dysfunction); LQTS, long‐QT syndrome; SCD, sudden cardiac death; S‐ICD, subcutaneous implantable cardioverter‐defibrillator; VT, ventricular tachycardia

Pooled data from two international studies (IDE and EFFORTLESS) revealed a relatively high proportion (70%) of S‐ICD implanted for primary prevention. In contrast, S‐ICD for primary prevention was indicated in only 58% of our nationwide registry collective: the predominant indications for implantation were aborted sudden cardiac death (27.4%), primary prevention in ischemic left ventricular dysfunction (23.9%), or dilated (non‐ischemic) CMP (12.8%); as well as previous TV‐ICD removal due to systemic infection (12.4%) (Figure 2). ^5^ Our panel's recommendations for implanting a non‐transvenous ICD system are summarized in Table 1. As clinical data are limited to nonrandomized studies, there was no “level of evidence A," according to the ESC classification for guidelines.

**TABLE 1:** Indications for S‐ICD implantation (Panel's recommendations based on ESC nomenclature)

Indications for S‐ICD implantation	Recommendation (level of evidence)
Missing or complicated transvenous access (especially in congenital heart disease)	I (B)
History of ICD extraction due to complications (especially after infections or thrombosis)	I (B)
Increased risk for infection (eg, diabetes, dialysis)	I (B)
Mechanical Tricuspid Replacement	I (C)
Severe tricuspid valve regurgitation	IIa (C)
Inherited arrhythmia syndromes or idiopathic VF	IIa (C)
Children and adolescents	IIa (C)
Frequent sportive activities	IIa (C)
Weak patient compliance (eg, after twiddler syndrome)	IIb (C)
Bridge to heart transplantation	IIb (C)
Indication for bradycardia pacing or cardiac resynchronization	III (C)
Expected need for anti‐tachycardia pacing (monomorphic VT)	III (C)
Failed pre‐implantation screening	III (C)

Abbreviations: ICD, implantable cardioverter‐defibrillator; S‐ICD, subcutaneous implantable cardioverter‐defibrillator; VF, ventricular fibrillation; VT, ventricular tachycardia.

Currently, there is no evidence based on randomized data showing any difference in terms of overall mortality, prevented sudden cardiac death, or the prevalence of (in‐) appropriate shocks between transvenous and subcutaneous ICD systems. However, the main advantages of S‐ICD systems in recent registries are the lower lead‐related complication rate (eg, systemic infections, venous thrombosis, lead fracture, or dislocation^2^) by using a mere subcutaneous instead of a transvenous lead with endomyocardial fixation, and its applicability in patients with specific anatomical conditions like limited venous access due to thrombosis or stenosis, congenital cardiovascular abnormalities or mechanical tricuspid valve replacement. In the recent 2017 AHA/ACC/HRS guidelines, an elevated risk for systemic infections (eg, diabetes mellitus, dialysis) was a class I‐A indication for preferring a *subcutaneous* ICD system.^7^ To this regard, the ongoing MADIT‐SICD trial will evaluate the clinical outcome of this therapy in diabetic patients with recent myocardial infarction.^8^ Pediatric and young patients might also benefit from this new technology, as subcutaneous leads are less affected by growth or frequent physical activity than transvenous leads.

Patients with IVF or channelopathies without expected need for ATP because of monomorphic VT, like catecholaminergic polymorphic VT, Long‐QT, or Brugada syndrome,^9^ may be appropriate candidates for S‐ICD as well. However, inappropriate shocks after T‐wave oversensing may be an issue in some arrhythmia syndromes (as discussed in the *Screening* section). Because of potential transvenous lead complications, further reasonable conditions for implantation of a subcutaneous system are frequent sportive activities, preexisting severe tricuspid regurgitation, weak patient compliance (eg, after twiddler syndrome), as well as bridging to heart transplantation when a long waiting period is expected, in order to avoid intravenous lead adhesions.

In case of failed preimplantation ECG screening, implantation of this system has to be avoided (see below). As an intermuscular position of the device is recommended (as discussed in the section 5.5: *Pitfalls of implantation*), patients with pronounced muscular weakness are no ideal candidates for this therapy. Since these devices do not provide intracardiac pacing, S‐ICD is contraindicated as first‐line therapy in patients requiring bradycardia pacing, cardiac resynchronization therapy (CRT), or ATP.^7^ In a recent ICD survey, these conditions were met in 45%, 26%, and 36%, respectively.^10^ Indeed, a subsequent upgrade to a transvenous ICD is possible whenever indicated, although the risk of an upgrade procedure has to be considered. In few cases, subcutaneous ICD were combined successfully with CRT‐, His bundle‐, or leadless pacemaker.^11^, ^12^, ^13^ In patients with left ventricular dysfunction following myocardial infarction, implanted loop recorder data revealed high‐degree AV blocks in 10% and sinus arrest in 5% after 2 years.^14^ This outcome was consistent with recent data, showing that a dual‐chamber ICD was definitely indicated in 11% after a mean follow‐up of one year after implantation.^15^

## 4 NEED FOR PREIMPLANTATION SCREENING

### 4.1 Automatic vs manual ECG screening

Like TV‐ICD, the S‐ICD monitors *RR* intervals. But in contrast to transvenous systems, S‐ICD lead sensing is based on far‐field ECGs. Additionally, the current QRS‐T morphology is matched to a morphology template for discrimination of supraventricular tachycardia (SVT).^16^ As this morphology analysis plays an important role in appropriate sensing, a QRS‐T morphology screening is recommended before implantation. The preimplantation screening should assure appropriate QRS detection and prevention of double‐counting due to T‐wave oversensing, which is the most important cause for inappropriate shocks in S‐ICD patients.^5^, ^17^

Initially, a manual ECG screening tool has been provided and showed 7.4% of patients not suitable for an S‐ICD.^18^ According to this study, hypertrophic cardiomyopathy (HCM), obesity, a bundle branch block, as well as a R/T ratio <3 in the ECG lead with the largest T wave were independently associated with screening failure. Recently, an automated screening algorithm (“*Vector Select”*) was integrated in the programming unit, where at least one vector ECG lead must be deemed acceptable for all tested postures: at least, supine and standing (or sitting) postures must be tested before implantation. Francia et al reported a higher acceptance rate with the automated screening tool, which was 23% more likely to predict the performance of the S‐ICD discrimination algorithm than the manual ECG screening tool.^19^

### 4.2 Need for exercise testing

In special collectives, such as HCM, Arrhythmogenic Ventricular Cardiomyopathy (ARVC), and Brugada Syndrome, automated screening during exercise testing should be considered.^20^, ^21^, ^22^, ^23^

In summary, we suggest that ECG screening should be performed with the automated screening tool in all patients prior to S‐ICD implantation. Exercise testing is specifically recommended in patients with ECG morphology changes, like in HCM, ARVC, and Brugada Syndrome.

## 5 IMPLANTATION ISSUES OF NTV‐ICD

### 5.1 General vs local anesthesia

In contrast to current practice for implantation of transvenous systems, general anesthesia (GA) is still standard of care for S‐ICD implantation procedures in many centers. Safe GA regimes have been published and offer sufficient analgesia as well as most periprocedural comfort for the patient and the implanter.^24^ Common risks are hemodynamic depression, postoperative nausea, and vomiting. Furthermore, postoperative analgesic management includes opioids, with an increased risk of morbidity especially in opioid‐naive patients.^25^ Thus, alternatives to GA should be pursued to keep the periprocedural risk as low as possible.

A retrospective multicenter study included 607 S‐ICD patients in 39 centers. Only 23% of implants were performed under GA and 77% under unconscious sedation or local anesthesia with sedation. Conscious sedation with propofol or midazolam, as well as local anesthesia was reported to be safe and efficient during intermuscular implantation.^26^

Finally, the combination of preoperatively administered oral acetaminophen and gabapentin with unconscious sedation and muscular plane block showed lower perioperative opioid consumption compared to GA plus muscular plane block.^27^ Of note, any multimodal anesthetic regime is complex and needs the expertise of an anesthesiologist.

### 5.2 Serratus and transversus thoracic plane blocks

As S‐ICD implantation can cause severe postoperative pain, regional anesthesia provides sufficient peri‐ as well as postoperative analgesia and can be performed in less than 10 minutes. The combination of two muscular blocks and local anesthesia are reported to be effective in S‐ICD procedures and provides almost 14 hours of analgesia. ^28^, ^29^

To this aim, *serratus anterior* plane block is an anesthetic technique targeting the S‐ICD pocket. Under ultrasound guidance, a local anesthetic (eg, lidocaine or bupivacaine) is infiltrated in the interfascial space between the *M. serratus anterior* and the *M. latissimus dorsi* about 20 to 30 minutes before skin incision.^28^

The *transversus thoracic* muscle plane block supports analgesia of the sternal region, where the lead is positioned. Again, a local anesthetic is injected under ultrasound guidance in the interfascial space between the *internal intercostal* and *transversus thoracic* muscle, in addition to local subcutaneous anesthesia before skin incision.^29^

### 5.3 Implantation techniques

S‐ICD implantation is a procedure without the risk for typical complications of transvenous systems (myocardial perforation, pneumo‐, or hematothorax). Device and lead are positioned either by definite anatomic landmarks or by skin markers confirmed by preoperative fluoroscopy, preventing intraoperative X‐ray exposure for both, patient and operator. The procedure is usually performed as a “three‐ “or “two‐ “incision technique (at least one incision for device pocket and xyphoid, respectively, with or without superior sternal incision).^30^ Even a “one incision” technique has been reported recently.^31^ The advantage of the “two incision” technique is mainly cosmetic. The main disadvantage of the “two incision” technique is the lacking suture, which holds the tip of the lead in the right layer and prevents erosion to the skin above. The “three incision” technique using a lead tip suture may prevent from the rare cases of twiddler syndrome in S‐ICD patients.^32^

The duration of an S‐ICD implantation is indeed more predictable than for transvenous systems, as the anatomical landmarks are well defined. Moreover, there are no anatomical obstacles within the cardiovascular system, as well as no necessity for obtaining optimal lead position based on specific measurements like pacing threshold or sensing.

Finally, the second generation of S‐ICD leads has an integrated sleeve for more stable lead position, in order to decrease the risk for lead dislodgement.

### 5.4 Shock efficacy testing vs PRAETORIAN score

Based on the randomized SIMPLE trial, shock efficacy testing (formerly performed as defibrillation threshold, DFT) is no more mandatory after implantation of TV‐ICD.^33^ As there are no controlled randomized data about the non‐inferiority of renouncing to shock efficacy testing after implantation of S‐ICD, this specific postimplantation procedure under unconscious sedation or GA is still recommended by the manufacturer. (***Recommendation I C***) To this regard, the ongoing randomized PRAETORIAN‐DFT trial is investigating the non‐inferiority of omitting shock efficacy testing in these patients.

Recently, the PRAETORIAN score has been published as a risk score for postimplantation shock efficacy, depending on fluoroscopically assessed device and lead position, as well as anatomical conditions.^34^ This score was retrospectively validated on clinical and computer modeling knowledge of determinants affecting the defibrillation threshold, like body mass index, sub‐coil and sub‐generator fat, as well as anterior positioning of the S‐ICD generator.

### 5.5 Pitfalls of implantation

As the learning curve for S‐ICD, implantation is steep and complication rates are low, incidence of suboptimal lead positioning is 0.8%.^35^ To ensure procedural success, incision and implantation sites should be marked on the skin prior sterile draping of the patient. According to manufacturer recommendations, the lead should be placed 1 cm to the left of the mid‐sternal line, attached (or at least close) to the fascia. When applying incise‐drapes for obese patients, it is recommended to use the “parachute technique” with an even coating of the skin in order to prevent deformation of the skin markers.

Compared to a true *subcutaneous* implantation, the “intermuscular” approach shows better cosmetic results and less device pocket complications and seems to improve DFT testing.^36^, ^37^ The interfascial plane between *M. serratus anterior* and *M. latissimus dorsi* offers a natural pocket for the device, allowing an atraumatic blunt dissection. As the muscular pocket minimizes the contact to the “isolating” subcutaneous fat tissue, intermuscular device position is crucial for optimal shock efficacy.

Tunneling the lead between xiphoid incision and device pocket has to be performed carefully. Continuous pressure on the tool‐handle, with the tip moving tangentially to the thoracic curvature, ensures subfascial lead placement with a safety distance from the intraperitoneal cavity.

Positioning of the S‐ICD lead on the fascial plane is of crucial importance, particularly in obese patients, the parasternal tunneling tool has to be inserted with its tip down, otherwise the lead coil might be implanted inappropriately into the poorly conducting subcutaneous fat tissue. In case of a superficial lead positioning, shock might be ineffective.^38^ Therefore, postoperative lateral chest fluoroscopy for confirmation of an optimal lead position is crucial for shock efficacy. Moreover, a chest X‐ray aims to rule out entrapped subcutaneous air bubbles around the lead, which might induce “baseline wandering” (undulating ECG baseline) and oversensing with the potential risk of inappropriate shocks in the first days after implantation.^39^, ^40^ Thus, before introducing the lead into the peel‐away‐sheath, it is highly recommended to flush the lumen with liquid (eg, saline solution) in order to avoid air bubble entrapment. Once the lead is placed and the sheath is peeled away, the incision site should be filled with liquid before massaging along the sternum from cranial to caudal, in order to evacuate the residual air. If air entrapment is recognized after implantation, vector reprogramming should be performed until air is resorbed after a few days. In case of initial lead misplacement (reported incidence of 3.5%), repositioning should be performed since it is safe and relevant for long‐term outcome. ^41^, ^42^ On the other hand, air bubble entrapment in the device pocket leads typically to a higher risk of shock failure during efficacy testing. This issue can simply be avoided by flushing the device pocket before wound closure.

In case of a damaged seal plug in the device header, fluid might intrude into the header and cause noise. As in all ICD devices, the seal plug has, therefore, to be handled with care, and must not be damaged with the torque wrench in order to avoid fluid or air leak into the device.^43^

## 6 PARTICULARITIES OF FOLLOW‐UP

### 6.1 Interrogation

Like all TV‐ICD devices, NTV‐ICDs are interrogated by a company‐specific programming unit after a wireless data connection over a wand. In S‐ICD devices, the following parameters can be interrogated: battery voltage, stored ECG episodes, current ECG (the programmed vector is displayed), as well as the programmed VT‐detection zones. Abnormal impedance is only indicated as an alert in case of lead fracture or insulation failure.

In case of failed interrogation, up to two ring‐magnets should be placed over the S‐ICD for resetting the device, in order to reenable the connection between the wand and the device.

### 6.2 Programming

In S‐ICD devices, only a few parameters can be programmed actively. First, two distinct zones for “shock” and “conditional shock” can be programmed for heart rates between 170 and 250 bpm. Tachycardia detection is based on 18 out of 24 beats, redetection on 14 out of 24 beats, and shock delivery is performed at 16 out of 24 beats during charging. In the same panel, extracardiac post‐shock pacing at 50 bpm for post‐shock asystole can be switched on/off. The maximum number of five shocks delivered within one episode is not programmable.

Three different vectors (primary, secondary, and alternative) can be programmed for optimal ECG detection. A change of programmed vector is recommended in case of documented over‐ or undersensing, in order to minimize the risk of either inappropriate or missing shocks. (***Recommendation I C***).

Finally, the integrated algorithms *SMART Pass* and *AF monitor* are integrated for better discrimination of SVT/VT and detection of paroxysmal atrial fibrillation, respectively.

### 6.3 Remote Monitoring

As there are only few parameters to be interrogated, remote monitoring might be the optimal mode of care for all patients with S‐ICD. (***Recommendation I C***) In patients with regular remote transmission, an on‐site interrogation is only necessary in case of shock delivery, detection of atrial fibrillation, lead impedance alert or depleted battery.

Regarding the *AF monitor*, the complete report on atrial fibrillation burden and episodes is only available by using remote monitoring.

### 6.4 General perioperative management

In order to prevent inappropriate shocks due to artifacts during surgical electro‐cautery, shock delivery has to be suppressed by fixing a ring‐magnet directly over the device. If the magnet is placed correctly, R‐wave synchronous beep‐tones should be heard 1 second after the magnet is applied. The magnet is acting on an integrated reed‐switch. If no beep‐tones are heard, or in case of inappropriate shocks despite applying one magnet, a second magnet should be placed in a stacked configuration over the first one. After the surgical procedure, the defibrillator function is restored by removing the magnet(s). As in patients with TV‐ICD, a deactivation and postoperative reactivation by using the programming unit is not necessary.^44^

### 6.5 MRI conditionality

The third generation of S‐ICD is full‐body MRI conditional for MRI scanners with a static magnetic field of 1.5 T. As these devices have no typical pacing function, the MRI‐related reprogramming simply consists in switching off the defibrillator function during the MRI scan. No serious complication during or after 1.5 T MRI has been documented yet.^45^

## 7 MANAGEMENT OF COMPLICATIONS

### 7.1 Infections

The main advantages of S‐ICD systems are the lower lead‐related complication rate by using a mere subcutaneous instead of a transvenous lead with endomyocardial fixation (eg, systemic infections, venous thrombosis, lead fracture, or dislocation).^2^

After a mean follow‐up of 3 years, rates of infection that required device removal were 2.4% in the EFFORTLESS registry, neither endocarditis nor systemic infections has been described till then.^41^, ^46^ Our national registry described a lower device‐related infection rate of 1.2% after a mean follow‐up of 1.7 years.^5^

Box exchange procedures are still a source for complications in cardiac device patients. Although lead dislodgement in S‐ICD seems to be very rare,^32^ risk of hematoma requiring evacuation might be comparable to TV‐ICD (0%‐1.6%).^47^

According to the 2009 HRS expert consensus on transvenous lead extraction, a reinfection of transvenous systems needing lead extraction is considered as a major complication, whereas a reinfection of a non‐transvenous system is a minor one.^48^ Remarkably, reinfection rates after TV‐ICD extraction in patients, who have consecutively received an S‐ICD, showed comparable reinfection rates when compared to patients with de novo S‐ICD implantation (1.3% vs 1.6%).^46^ In a more recent series comparing post‐TLE implantation of both ICD systems, the overall rate of complications was significantly lower in the S‐ICD group when the device was positioned in a sub‐ or intermuscular pocket.^49^

S‐ICD pocket infection should be managed as any other device infection; however, risk for device‐related systemic infection is probably significantly lower than in TV‐ICD devices.^46^ Extractions of subcutaneous systems can be performed in any center without cardiac surgery support. In order to avoid (re‐) infection during box exchanges, application of an antibiotic envelope might provide protection as in transvenous systems,^50^, ^51^ whereas there are no data for S‐ICD to this regard.

### 7.2 Inappropriate shocks

Discrimination of the QRS complex from the P‐ and T‐waves is crucial and needs exact ECG screening before implantation, as well as integrated discrimination algorithms like *SMART Pass*.^52^

The prevalence of inappropriate shocks by the S‐ICD was analyzed prospectively in the studies IDE^53^ and EFFORTLESS^46^: in a pooled analysis including 882 S‐ICD patients, the rate of inappropriate shocks in the S‐ICD was 13% after 3 years, but decreased significantly over time.^35^ In our national registry, inappropriate shocks occurred in 5.2% within a follow‐up of 1.7 years.^5^ In both studies, the majority of inappropriate therapies were due to oversensing of different non‐ventricular signals, mainly T‐wave oversensing. In contrast, inappropriate therapies in TV‐ICD are mainly driven by SVT. In a recent meta‐analysis comparing TV‐ICD and S‐ICD, the prevalence of inappropriate shocks showed no significant difference (7% vs 9%).^54^ Since inappropriate shocks reduce patients' quality of life, there is still a need for improvement in technology to reduce noise‐ or T‐wave oversensing in S‐ICDs. In contrast, the better performance of S‐ICD in SVT is most likely due to the software's reliable tachycardia discrimination.

In case of serial inappropriate shocks, up to two magnets should be placed over the S‐ICD in the acute phase. Depending on the underlying cause, reprogramming of the vector in case of over‐ or undersensing, or surgical revision of the lead has to be performed in case of dislodgement or abnormal impedance. If inappropriate shocks occur in a first‐ or second‐generation device, a software update has to be taken into account, if not done yet.

### 7.3 Ineffective shocks

In the pooled three‐year follow‐up data of IDE and EFORTLESS, efficacy of the first shock was 90%, whereas the “final” shock episode was efficient in 98%.^35^ In our national S‐ICD registry, these rates even reached 96% and 100%, respectively.^5^ If a patient experiences multiple ineffective ICD shocks, the incessant VT may lead to acute heart failure, associated with a high mortality. Since the number of implanted devices analyzed postmortem is low, there is no reliable information about the amount of S‐ICD therapies delivered without terminating life‐threatening arrhythmia.

In case of recurrent ineffective shocks, a change of the programmed vector should be considered before surgical revision of the implanted system. In the latter case, the criteria of the PRAETORIAN score should be taken into consideration.^34^ If anatomically suitable, switching to a transvenous system might be a further alternative in these rare cases.

The recently presented PRAETORIAN trial was the first randomized trial showing that S‐ICD therapy is non‐inferior to TV‐ICD regarding ICD‐related adverse events, with a comparable all‐cause mortality in both groups. Especially, lead‐related complications were significantly lower in the S‐ICD group.^55^ In the meta‐analysis mentioned above, both ICD systems performed equally regarding the efficacy of appropriate shocks.^54^ Therefore, shocks delivered by the S‐ICD showed high efficacy, but an improvement of mortality by this novel device is still to be shown in an ongoing randomized trial.

## 8 COST EFFECTIVENESS

Although more expensive, S‐ICD were associated with a relative risk reduction for device‐related complications of 70% compared to TV‐ICD. In this propensity matched case‐control study, total mean costs (including complication‐related costs) were significantly lower in patients with a subcutaneous system over a period of 5 years.^56^

To date, Austria is the only country worldwide with a separate reimbursement for the implantation of S‐ICD devices, which is 22% higher than the one for TV‐ICD without CRT. Taking into consideration the specificities of device implantation and management, a specific reimbursement should be sought by the authorities, in order to enable the implantation of S‐ICD when indicated.

## 9 FUTURE PERSPECTIVES

### 9.1 Subcutaneous ICD

For the upcoming fourth generation, parallel processing of all three vectors is expected to provide improved sensing properties and increaing the accuracy of discrimination, thus minimizing the number of screening failures and overcome the need of vector reprogramming. As pulse generator size is still an issue in children, adolescents, or patients with low body mass index, the development of smaller device cans is expected in future. As mentioned above, an intermuscular device implantation already enabled efficient shocks using lower energies in the DFT testing.^37^ Finally, one future concept is to pair a leadless pacemaker in VVI mode with the S‐ICD from the second generation, in order to provide ATP and (post‐shock) bradycardia pacing for patients who are expected to benefit or have a respective indication.

Currently, the lack of ATP is still a major issue for a better acceptance of the S‐ICD among cardiologists. However, ATP should be programmed more conservatively, since the randomized MADIT‐RIT trial revealed a higher mortality associated with appropriate ATP therapies in the “conventional” (empiric) ICD programming arm.^57^ One explanation for this finding might be a significantly higher mortality in patients with accelerated VT following appropriate ATP intervention.^58^

### 9.2 Extravascular‐ICD

After a promising feasibility study,^3^ the first patient implant of an EV‐ICD system with a novel S‐shaped retrosternal lead in August 2018 marks the beginning of the ongoing pilot study.^59^, ^60^ If the predefined efficacy and safety outcomes of the pilot study will be met, a pivotal trial will be launched later within the next year.

### 9.3 Implantable subcutaneous string defibrillator

The novel implantable subcutaneous string defibrillator (ISSD) is a flexible, string‐shaped, and rechargeable non‐transvenous ICD. The ISSD device is comprised of a sternal coil, a left‐lateral coil (each 10 cm long), and a 4 cm long cylindrical tube in between, called the “active segment.” First experiences were reported in 22 patients. The average implant time was 20 minutes and results include an average DFT of 25.8 ± 10.7 J in successfully screened patients.^61^ Possible benefits of this device are its minimally invasive implantation via small incisions, no need for a classical device pocket, aesthetic appearance, and its rechargeability (1 hour once a year wireless recharge designed for 10 years longevity).

## 10 CONCLUSION

This consensus of our national expert panel gives an overview of potential indications for the implantation of non‐transvenous ICD systems, and provides specific recommendations for implantation, follow‐up, and complication management in patients with S‐ICD. Regarding particular issues like the necessity for shock efficacy testing, or the clinical outcome in comparison to TV‐ICD, randomized data are expected in the near future.

## CONFLICT OF INTEREST

The authors declare no potential conflict of interest.
